# Adolescent social stress increases anxiety-like behavior and ethanol consumption in adult male and female C57BL/6J mice

**DOI:** 10.1038/s41598-018-28381-2

**Published:** 2018-07-03

**Authors:** M. J. Caruso, L. R. Seemiller, T. B. Fetherston, C. N. Miller, D. E. Reiss, S. A. Cavigelli, H. M. Kamens

**Affiliations:** 1 Department of Biobehavioral Health, Pennsylvania State University, University Park, PA 16802 USA; 2 The Huck Institutes for the Life Sciences, Pennsylvania State University, University Park, PA 16802 USA; 3 Present Address: Department of Systems Pharmacology and Translational Therapeutics, Perelman School of Medicine, University of Pennsylvania, Philadelphia, PA 19104 USA

## Abstract

Exposure to social stress is an important risk factor for comorbid affective disorders and problem alcohol use. To better understand mechanisms involved in social stress-induced affective disorder and alcohol use co-morbidity, we studied the effects of adolescent social stress on anxiety- and depression-like behaviors and binge-like ethanol consumption. Male and female C57BL/6J mice were exposed to chronic variable social stress (CVSS) or control conditions throughout adolescence (postnatal days, PND, 25–59) and then tested for anxiety-like behavior in the elevated plus maze and a novel open field environment, or depression-like behavior using the forced swim test on PND 64–66. Mice were then tested for binge-like ethanol consumption using the Drinking-in-the-Dark model. Male and female mice exposed to adolescent CVSS had increased adult anxiety-like behavior and increased locomotor adaptation to a novel environment. Further, CVSS mice consumed significantly more ethanol, but not saccharin, than controls. Despite group differences in both anxiety-like behavior and ethanol consumption, there was no relationship between these outcomes within individual mice. These data suggest that exposure to adolescent social stress is an important risk factor for later alcohol use and affective behaviors, but that social stress does not necessarily dictate co-morbidity of these outcomes.

## Introduction

Alcohol use disorders (AUD) are common in the United States with a total lifetime prevalence of 30%^1^. Affective disorders, including anxiety disorders and major depression, also frequently co-occur with AUDs and chronic adolescent stress exposure is a risk factor for the development of both^2^. Adolescent stress may promote vulnerability to comorbid AUDs and affective disorders through its impact on common underlying biological factors^3^. Preclinical models have provided valuable insight into the neurobiology of adolescent stress-induced vulnerability to AUDs, affective disorders, and their comorbidity, but have relied heavily on male rodents^4^. Given that clinical studies have associated early life stress with higher rates of comorbid AUDs and affective disorders in females than males^5,6^, additional preclinical research on sex differences in vulnerability to stress-related AUDs and affective disorder comorbidity is necessary.

Because social stressors are among the most potent and prevalent stress stimuli that humans can experience^7^, rodent models of social stress may provide clinically relevant insight into factors underlying the adverse impact of adolescent stress on comorbid AUDs and affective disorders^2,8^. Accordingly, adolescent social isolation, social instability, and repeated social defeat were found to increase voluntary ethanol consumption or self-administration in male rats and mice^9–12^. These same adolescent social stress models also elicited changes in anxiety/depression-like behaviors^4,10,13–16^. Two important limitations of the prior work are that current models have focused on male animals or failed to observe consistent findings in females and, thus, do not provide insight into sex differences in susceptibility^4,9–12,17^. Furthermore, few studies have investigated the relationship between stress-induced changes in ethanol consumption and anxiety - or depression-like behavior within the same animals. Therefore, it is difficult to generate hypotheses regarding neurobiological mechanisms that may underlie stress-induced AUD and affective disorder comorbidity, and potential sex differences in these mechanisms.

We have recently developed an adolescent chronic variable social stress protocol (CVSS) that has proved useful for studying adolescent stress-induced addiction- and anxiety-related phenotypes in male and female inbred mice^18–20^. For example, the protocol, which involves repeated cycles of individual housing and with novel social partners, enhanced acute nicotine-induced locomotor activity during late adolescence and reduced adult nicotine consumption in male BALB/cJ mice^18^. Further, in adult male and female C57BL/6J mice, CVSS increased anxiety-like behavior on the elevated plus-maze (EPM) and evoked sex-specific alterations in synaptic excitability of prefrontal cortex (PFC) and nucleus accumbens (NAC) neurons in adult male and female C57BL/6J mice^19^. These findings are intriguing because the development of anxiety-, depression-, and addiction-like behaviors is believed to involve synaptic modifications within the PFC and NAC among other corticolimbic regions^21^. Moreover, this circuit is subject to several convergent mechanisms of stress-, nicotine-, and ethanol-evoked synaptic plasticity that could contribute to heightened drug susceptibility^22^. As such, CVSS may be a useful model of adolescent stress-induced susceptibility to AUD and affective disorder comorbidity in both male and female mice.

Here we sought to replicate and extend prior findings in male and female C57BL/6J mice by investigating the influence of CVSS on binge-like ethanol consumption using the Drinking-in-the-Dark (DID) paradigm, affective-like behaviors, and locomotor exploration. Specifically, we examined anxiety-like behavior in the EPM, depression-like behavior in the forced swim test (FST), and locomotor adaptation in the open field test (OFT). These specific behaviors were selected because stress-induced changes are associated with increased ethanol consumption^4^. We hypothesized that CVSS would increase binge-like ethanol consumption, increase anxiety- and depression-like behavior, and impair locomotor adaptation in both males and females. We also hypothesized that, within individuals, greater levels of anxiety/depression-like behavior and lower levels of locomotor adaptation would be correlated with increased binge-like ethanol consumption.

## Results

**Figure 1:**
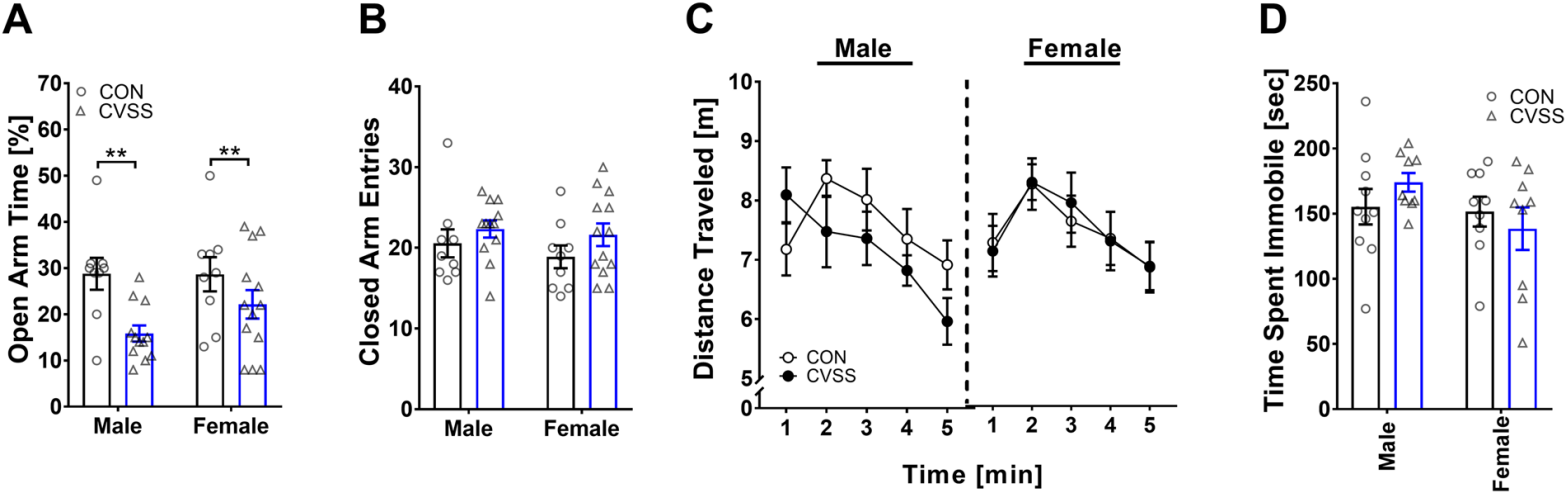
Adolescent CVSS increased anxiety-like behavior in adult males and females. Data (mean ± SEM) represent (**A**) percent time spent on the open arms of the EPM, (**B**) number of closed arm entries on the EPM, (**C**) total distance traveled in the OFT, and (**D**) time spent immobile in the FST. Significant main effect of stress condition: **p < 0.01. (n = 9–15/group).

Adolescent CVSS increased adult anxiety-like behavior and increased habituation to a novel environment in the EPM and OFT, respectively (Fig. 1). Consistent with our previous findings, CVSS mice spent significantly less time on the open arms of the EPM relative to CON mice (Fig. 1A; main effect of stress condition: F_1,38_ = 9.42, *p* < 0.01). CVSS mice also entered the open arms of the EPM significantly less than CON mice (main effect of stress condition: F_1,38_ = 9.23, *p* < 0.01; 37.1 ± 1.6% vs. 44.9 ± 1.9%, respectively). There were no significant effects of sex or sex x stress condition interactions for time on and entries into the open arms. There were no significant effects of sex or stress condition on closed arm entries in the EPM (Fig. 1B). In the OFT, there was a significant main effect of time (F_4,128_ = 10.34, *p* < 0.001) and a significant interaction of time x stress condition (F_4,128_ = 3.39, *p* < 0.05) for distance traveled, but post hoc analyses failed to identify significant differences between CVSS and CON mice at any given time point (Fig. 1C). There were no significant main effects of sex or stress condition for total distance traveled across the five minutes. However, adolescent CVSS altered habituation to a novel environment; CVSS mice exhibited significantly greater locomotor adaptation (change in locomotion during the first and fifth minute of OF exposure) relative to CON mice (main effect of stress condition: F_1,31_ = 6.54, *p* < 0.05; 1.7 ± 0.38 m vs. 0.3 ± 0.38 m, respectively). Finally, although males spent more time than females in the center of the open field (main effect of sex: F_1,31_ = 5.13, *p* < 0.05; 9.9 ± 0.7% vs. 7.7 ± 0.7%, respectively), there was no significant effect of stress condition or sex x stress condition interaction on locomotor adaptation. There were no significant effects of sex or stress condition on time spent immobile in the FST (Fig. 1D).

**Figure 2:**
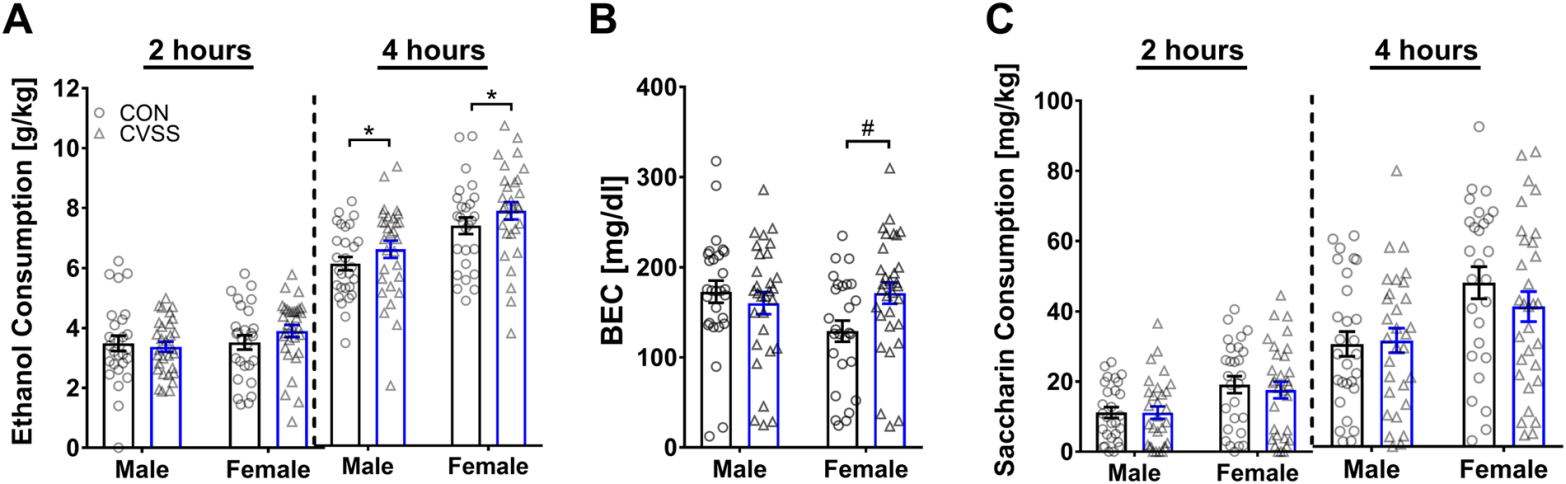
Adolescent CVSS increased binge-like ethanol consumption in adult males and females. Data (mean ± SEM) represent (**A**) ethanol consumption, (**B**) blood ethanol concentrations (BEC), and (**C**) saccharin consumption. Significant time x stress condition interaction: *p < 0.05. Significant sex x stress condition interaction: ^#^p < 0.05. n = 9–15/group. Data for males and females are combined for ethanol and saccharin consumption.

Binge-like ethanol consumption was greater in mice exposed to adolescent CVSS relative to CON mice (Fig. 2A). Analyses were performed separately for ethanol consumption at 2 and 4 h due to a significant time x sex interaction (F_1,111_ = 19.11, *p* < 0.0001). At 2 h, there were no significant effects of sex or stress group or the interaction of these factors (Fig. 2A). At 4 hr, CVSS mice consumed significantly more ethanol than CON mice (Fig. 2A; main effect of stress condition: F_1,110_ = 5.15, *p* < 0.05) and females consumed significantly more ethanol than males (main effect of sex: F_1,110_ = 14.75, *p* < 0.001; 7.8 ± 0.2 g/kg vs. 6.8 ± 0.2 g/kg, respectively).

Adolescent CVSS increased blood ethanol concentration (BEC) in females but not males (Fig. 2B). Overall, ethanol consumption in the DID paradigm resulted in pharmacologically relevant BECs (mean BEC: 159.1 ± 6.1 mg/dl) and there was a significant positive correlation between ethanol consumption at 4 h and BECs in male (*r* = 0.38, df = 55, *p* < 0.01) and female mice (*r* = 0.39, df = 56, *p* < 0.01). Additionally, adolescent CVSS increased BECs in a sex-dependent manner (sex x stress condition interaction: F_1,111_ = 4.80, *p* < 0.05); CVSS females had higher BECs than CON females (Fig. 2B; Tukey’s HSD, *p* < 0.05), and there was no difference in BEC between CVSS and CON males (Fig. 2B).

To determine if the increase in ethanol consumption was specific to alcohol, we examined the effect of CVSS on saccharin consumption in the DID paradigm. Saccharin consumption was not affected by adolescent CVSS (Fig. 2C). Analyses were performed separately for saccharin consumption at 2 and 4 h due to a significant time x sex interaction (F_1,111_ = 5.79, *p* < 0.05). Overall, females consumed more saccharin than males at 2 h (main effect of sex: F_1,111_ = 13.16, *p* < 0.001; 17.9 ± 1.2 g/kg vs. 11.6 ± 1.2 g/kg, respectively) and 4 h (main effect of sex: F_1,111_ = 9.74, *p* < 0.01; 39.3 ± 2.3 g/kg vs. 28.7 ± 2.3 g/kg, respectively). Finally, there were no significant associations between saccharin consumption and ethanol consumption at 2 h or 4 h in either male of female animals.

Correlation analyses were performed to evaluate the relationship between binge-like ethanol consumption and anxiety- and depression-like behaviors. When analyzed separately in males and females, there were no significant associations between anxiety- and depression-like behaviors and binge-like ethanol consumption (data not shown).

## Discussion

This study investigated the influence of adolescent chronic variable social stress (CVSS) on adult binge-like ethanol consumption and anxiety- and depressive-like behaviors in inbred male and female C57BL/6J mice. Our results show that CVSS increased binge-like ethanol consumption in both male and female mice. This effect was specific to ethanol as there was no effect of CVSS on saccharin consumption. CVSS also increased anxiety-like behavior on the EPM, replicating our previous findings^19^, and increased locomotor adaptation to a novel environment. Depression-like behavior, as measured with the FST, was not altered by adolescent CVSS. Overall, these findings contribute to the growing body of literature demonstrating that exposure to adolescent social stress can increase risk for later affective disorders and AUDs^4,9–12^. In particular, the results contribute important information on long-lasting effects of social stress in male and female rodents.

We found that adolescent social stress exposure increases binge-like ethanol consumption. We previously found that adolescent CVSS altered nicotine responses in male BALB/cJ^18^, but not in C57BL/6J^19^, mice suggesting that CVSS can influence addiction-related behaviors, but these effects may be dependent upon genetic background. Consistent with the current findings, adolescent social isolation has been shown to increase adult ethanol consumption in male rats and mice^4,9–12^. Although the preponderance of evidence suggests that social stress exposure increases ethanol consumption in male rodents, results with females are mixed. For example, as in the current study, transient increases in ethanol consumption were observed following adolescent social isolation in some studies, whereas others have observed no differences in ethanol intake following social isolation or social instability stress^9,11,17^. Interestingly, we saw no significant differences in ethanol consumption at 2 hours, but by 4 hours a difference was observed. This suggests that group differences in intake may emerge over longer periods of ethanol exposure, a finding consistent with other studies^23,24^. Moving forward it will be important to understand the biological mechanism(s) that mediate this combination of sex-, genotype-, and time-specific effects of adolescent social stress. Furthermore, future studies should determine how long these CVSS-induced behavioral changes last. For example, the effect of social isolation on ethanol consumption is transient in female Long Evans rats^17^, but longer lasting in males^10^. Our results suggest increased consumption in both male and female C57BL/6J mice, but it is unclear whether this increase persists, or whether it persists in one sex but not the other.

Based on prior human and rodent study results, we hypothesized that there would be a positive association between anxiety-related behavior and ethanol consumption within individuals^2,8^. Interestingly, we did not observe significant associations between individual differences in ethanol consumption and affective-like behaviors. Previous evidence in male rats has shown a positive correlation between anxiety-like behavior on the EPM and ethanol consumption following adolescent social isolation^25^, but this relationship was not observed in female rats undergoing the same procedure^17^. In contrast, opposite results have been reported with the light-dark box, another measure of anxiety-like behavior, where rats that spent more time in the light portion of the chamber (i.e., a low anxiety phenotype) consumed greater amounts of ethanol^17^. Given these discrepant results, it is difficult to draw strong conclusions but it is possible that social stress affects multiple brain regions, some involved in anxiety and others related to ethanol consumption. One important limitation to our current study was the use of inbred C57BL/6J mice. This strain was chosen because it voluntary consumes a large amount of alcohol. However, it is possible that this masked potential relationships between anxiety-like behavior and drug consumption. Future studies could use strains of mice that are moderate ethanol consumers or outbred mice that may exhibit more variable behavioral responses. We have previously shown that CVSS alters synaptic transmission in the PFC and NAC of C57BL/6J mice^19^. These regions were examined because of their implication in affective-like behaviors and drug responses^21^ and may, therefore, contribute to the changes in anxiety-like behavior and ethanol consumption observed in the current study.

Exposure to adolescent social stress increased anxiety-like behavior on the EPM and increased locomotor adaptation to a novel environment in the OFT with no effect on total locomotor activity or time spent in the center of the arena. These findings replicate our prior study demonstrating that adolescent exposure to CVSS can lead to increased anxiety-like behaviors (i.e., reduced time on open arms of the EPM)^19^, but suggest that this phenotype may not extend to another common anxiety-like behavior (e.g., the OFT). Previous studies have shown that adolescent social stress increases anxiety-like behavior in the EPM with adult rats and mice^4,10,13,15^. In addition to replicating anxiety-like effects on the EPM, locomotor adaptation to a novel environment was measured in the OFT. Prior work has found that altered locomotor adaptation to a novel environment after exposure to social stress correlates with several anxiety-like behaviors, including decreased open arm time on the EPM, and can be reversed with anxiolytic and antidepressant treatments^14–16^. Notably, these studies have demonstrated both increased^15^ and decreased^14^ locomotor adaptation, with no change in general locomotor activity, following adolescent social instability stress. As such, non-specific alterations in the locomotor responses to novelty, not an increase or decrease *per se*, may reflect a stress-induced anxiety-like phenotype. Given our EPM results it is possible that the effects of CVSS on locomotor adaptation represent another anxiety-like state, but this seems unlikely given that there was no change in the time mice spent in the center area during the OFT. A detailed analysis of the complexity in this relationship is beyond the scope of the current study.

Although major depressive episodes are commonly preceded by events of chronic stress^21^, our present study found no effect of exposure to adolescent social stress on adult depression-like behavior in the FST. This finding replicates our prior work with male BALB/cJ mice^20^. Further, adolescent female, but not male, rats exposed to social instability stress displayed a transient increase in immobility in the FST during adolescence that diminished in adulthood^26^. Together, these data suggest that females are more susceptible to the depression-like effects of adolescent social stress and that these effects might be short lived. In the current study, it is possible that we missed a transient adolescent increase in depression-like behavior that resolved in adulthood. Alternatively, genetic variability has a strong influence on depression-like behavior^27^. Therefore, the results from prior studies that utilized genetically variable outbred rats^26^ or a different inbred mouse strain (i.e., BALB/cJ)^20^ may not generalize to C57BL/6J mice. Interestingly, we have observed a similar disparity when investigating the effects of CVSS on EPM behavior in BALB/cJ and C57BL/6J mice^18,19^.

There are limitations to consider when interpreting the results of this study. First, depression-like behavior was only evaluated with one behavioral test. Future studies should evaluate the effects of CVSS on additional depression-like behaviors such as the sucrose preference test, generally thought to model anhedonia which is a core symptom of depression^21^, or immobility behavior in the tail suspension test (TST). Although seemingly analogous, unique biological substrates appear to underlie immobility in the FST and TST^28^. Furthermore, we found that female  BALB/cJ mice exposed to CVSS exhibited decreased sucrose preference with no effect on immobility in the FST^20^. Another limitation to the current study is that estrous cycle was not monitored in females. In rats, estrous cycle can moderate ethanol consumption^29,30^ and the effects of adolescent social instability stress on female anxiety-like behavior^26^. Future research will be necessary to determine the impact of estrous cycle variation on the behavioral effects of CVSS.

The comorbidity in humans between affective disorders and AUDs calls for a better understanding of the neurobiology underlying these disorders. This study demonstrates that exposure to social stress during adolescence increases binge-like ethanol consumption and anxiety-like behavior, but these behaviors were not associated within individuals. Future studies should focus on determining the factors that predict correlated changes in affect-related behaviors and ethanol consumption following adolescent stress to better understand this comorbidity in both sexes.

## Materials and Methods

### Animals

Male and female C57BL/6J mice (The Jackson Laboratory, Bar Harbor, ME) were bred at The Pennsylvania State University. A total of 116 mice (55 females and 61 males) from 22 litters were used. Estrous cycle was not monitored in the female mice. Pups remained with the dam until weaning on postnatal day (PND) 21 when they were housed with 3–4 same-sex cagemates in polycarbonate cages (28 cm × 17 cm × 12 cm) with corncob bedding in a temperature and humidity-controlled vivarium. Mice were maintained on a reverse 12-h light-dark schedule (lights on at 10:00 h) with *ad libitum* food and water. All procedures followed the National Institutes of Health guide and were approved by the Pennsylvania State University IACUC committee.

### Chronic Variable Social Stress (CVSS) and experimental design

The adolescent CVSS paradigm was performed as previously described^18,19^. Male and female mice were randomly assigned to the CVSS or control (CON) group. Littermates were evenly distributed between groups to control for litter effects and all mice remained housed in the same colony room. Beginning on PND 25, CVSS mice were individually housed for 3 days followed by 4 days of re-socialization with 1–2 unfamiliar same-sex cagemates (i.e., social reorganization). This CVSS cycle was repeated until PND 59. This age-range encompasses the early to late adolescent stages of development, based on age-specific behavioral, neurobiological, and pubertal changes^31^. The CON mice remained with their original cagemates throughout the experiment. To control handling and husbandry between groups, all CON mice were placed in clean cages twice per week. On PND 59, CVSS mice were re-housed with their original cage-mates from weaning, where they remained throughout behavior testing. Adult anxiety-like, exploratory, and depression-like behaviors were assessed 5–7 days after the conclusion of CVSS using the EPM, OFT, and FST, respectively (see Table 1). Separate groups of mice were assigned to each behavioral test to avoid any influence of prior experience on test performance. All behavioral tests except DID (see Section 2.6.) were conducted in a room that was separate from the colony room between 9:00–13:00 h. Mice were allowed to habituate to the behavior room for at least 1 h prior to testing.

**Table 1:** Overview of experimental design.

Group	Subjects	Procedures
1	CON = 10 M & 9 FM	EPM (PND 64–66) → Ethanol DID (PND 74–77) → Saccharin DID (PND 88–91)
	CVSS = 12 M & 13 FM	
2	CON = 9 M & 9 FM	OFT (PND 64–66) → Ethanol DID (PND 74–77) → Saccharin DID (PND 88–91)
	CVSS = 9 M & 9 FM	
3	CON = 9 M & 10 FM	FST (PND 64–66) → Ethanol DID (PND 74–77) → Saccharin DID (PND 88–91)
	CVSS = 9 M & 9 FM	

CON = control; CVSS = chronic variable social stress, DID =  Drinking-in-the-Dark; EPM = elevated plus-maze; FST = forced swim test; OFT = open field test; PND = postnatal day.

### Elevated plus-maze (EPM)

The effect of adolescent CVSS on anxiety-like behavior was measured in the EPM^18,19^. The EPM consisted of two open (30 × 5 cm) and two closed (30 × 14.5 × 5 cm) Plexiglas arms elevated 42 cm above the ground. Testing occurred under dim red lights (~30 lx). Each mouse was recorded by an overhead camera and behavior was scored for 5 min by an automated video tracking system (ANY-maze v.4.6, Stoelting, Wood Dale, IL, USA). The percent time spent on open arms (Time on open arms/[Time on open arms + time on closed arms] × 100) and percent open arm entries (Open arm entries/[Open arm entries + Closed arm entries]) were used as measures of anxiety-like behavior and the number of closed arm entries was used as a measure of general locomotor activity^32,33^.

### Open field test (OFT)

The effect of adolescent CVSS on locomotor adaptation to a novel environment was measured in the OFT. This measure was chosen based on prior studies in which adolescent C57BL/6 and CD1 mice exposed to social stress displayed alterations in locomotor adaptation that were prevented by treatment with antidepressant or anxiolytic drugs^13–16^. The OFT arena (60 × 60 × 30 cm) was constructed from white Plexiglas and illuminated by dim red lights (30 lx). General locomotor activity (e.g., distance traveled) was recorded for 5 min by an overhead camera and analyzed using an automated video tracking system (ANY-maze v.4.6, Stoelting, Wood Dale, IL, USA). The primary dependent variables were total distance traveled, locomotor adaptation (cm), calculated as [distance traveled during min 1 – distance traveled during min 5], and percent time spent in the center of the open field.

### Forced swim test (FST)

The effect of adolescent CVSS on depression-like behavior was measured using the FST as described previously^20^. Briefly, mice were placed into an inescapable glass beaker (24.5 × 18.5 cm) filled with water (25–27 °C) for 6 min. The primary dependent variable, time spent immobile (sec), was defined as floating and making only those movements necessary to keep the head above water. Time spent immobile was measured during the last 4 min of the test^34^. A trained observer who was blind to the experimental condition scored FST behaviors.

### Drinking-in-the-Dark (DID)

The effect of adolescent CVSS on binge-like ethanol consumption was evaluated using a standard 4-day DID paradigm^35,36^. Ethanol (20% v/v) was prepared from ethyl alcohol (200 proof; Koptec) diluted with tap water. Briefly, mice were individually housed on PND 66 for 1-week prior to DID with free access to food and water to acclimate to the testing environment. On days 1–3, water bottles were removed 3 h into the dark cycle and mice were given access to a single bottle containing 20% ethanol for 2 h. Binge-like ethanol consumption was assessed on day 4, when mice were given access to 20% ethanol for 4 hours. The volume of fluid in the bottle (read to the closest 0.1 ml) was recorded immediately upon placement and at 2 h and 4 h. Immediately after the 4 h test, ~10–20 μl of blood was collected by tail nick, and blood ethanol concentrations (BEC) were measured using an Analox AM1 Analyzer (Analox Instruments, Lunenburg, MA). To ensure that the effect of adolescent CVSS was specific to ethanol, mice were retested with same procedure, but had access to 0.033% saccharin instead of ethanol^37,38^. For ethanol and saccharin consumption, two control cages with no mouse were handled with the same procedure as experimental cages. The average leakage from these control cages was subtracted from the drinking values obtained for the experimental animals. The primary dependent variables obtained were ethanol consumption (g/kg), saccharin consumption (mg/kg), and BEC (mg/dl).

### Statistics

All statistical analyses were performed in R (v3.3.2). Analyses of results from the EPM, OFT, FST, and BECs were analyzed using analysis of variance (ANOVA). A repeated measures ANOVA was used to analyze data from DID behaviors and total distance traveled in the OFT. All data were tested for normality and homoscedasticity and were found to meet the assumptions for parametric analyses. One outlier was detected using Grubb’s outlier test and removed from the analyses (DID ethanol consumption: 1/112)^39^. Independent factors included stress condition, sex, and time. In all statistical models, continuous litter means were included as covariates to control for litter effects. Whenever a significant main effect or interaction was identified post hoc analyses were performed using Tukey’s HSD. To test the relationship between ethanol consumption and behavior in the EPM, FST, or OFT within individual mice, partial correlations, controlling for litter, were calculated separately for males and females in each experiment. An α < 0.05 was considered significant for all statistical analyses including post hoc comparisons. Data are presented as mean ± standard error of the mean (SEM).

### Data availability

The datasets generated during and/or analyzed during the current study are available from the corresponding author on reasonable request.
